# Epidemic Erysipelas, Called “Black Tongue”

**Published:** 1844-05

**Authors:** 


ILLINOIS

MEDICAL & SURGICAL JOURNAL.

VOL. I. MAY, 1844. NO. 2.

Epidemic Erysipelas, called "Black Tongue."—This disease has for several years been prevailing in various sections of the Union. Appearing at different times, at points widely separated from each other, and with a fearful mortality, it has attracted considerable attention. The principal accounts of it which have been given to the public, are by Charles Hall, M. D., of Burlington, Vt., and George Dexter, M. D., of Lancaster, N. H., in a joint paper communicated to the Amer. Jour. of Jan. '44, and by Dr. George Sutton, of Aurora, Ia., in the Western Lancet, Nov. 1843. We shall take the privilege of arranging from these and other accounts, the most prominent features of this disease, and its treatment.

The first appearance of Epidemic Erysipelas, so far as we can learn, was in the summer of 1841, in some parts of Canada. At Lancaster, N. H., several cases occured late in the fall of 1841. In the spring of 1842, it appeared at different points in the northern and middle sections of Vermont and New Hampshire; and during the whole of that year, it prevailed to a greater or less extent in that district. The line of its progress, Dr. Hall represents as "irregular and erratic," he indicates a "section of country, from the Canada line following the course of the Connecticut river southwardly, a distance of a hundred miles, westwardly, from the banks of the same river, to the borders of Lake Champlain, and eastwardly, to the State of Maine," as that in which the disease manifested itself in the most malignant form.

Dr. Sutton records the appearance of the epidemic in Ripley county, in November, 1842. "It commenced three miles east of Napoleon, and gradually extended in a south-easterly direction, over a section of country ten to fifteen miles in width, and about thirty in length." During the winter of 1842, notice appeared in the public papers of an epidemic prevailing on the Illinois river, called the "black tongue;" also of a similar or identical disease in some parts of Missouri.

During the last winter, (1843, '44,) the same epidemic prevailed at Michigan City, Ia., Our accounts of the disease occurring at this point have, as yet, been verbal,* but sufficient to remove all doubt of an identity with those already mentioned.

A communication in the Columbia, (Tenn.) Observer, (March 7, '44,) mentions an epidemic erysipelas as prevailing in that town. The symptoms characterizing it do not show any material variation from that prevailing in other sections of the country.

A late newspaper account represents the "black tongue" as "raging with great violence in Augusta, Kentucky.

Such have been the principal points at which the disease has made its appearance. The irregularity of its progress, and the possibility of its still extending to other places, render it desirable that the profession should inform themselves upon the subject.

The symptoms of the disease in the different sections enumerated, are, in many respects, strikingly similar. We cannot do better than quote from the different observers.

Drs. Hall & Dexter describe them as follows:

"With few exceptions, for two or three months after the first appearance of the erysipelas, there was great uniformity in the early symptoms and manner of attack. The disease was ushered in by many of the premonitory symptoms of pyrexia; sore throat more or less severe; enlarged tonsils and submaxillary glands; difficult deglutition, and sometimes painful respiration, attended with lassitude, pain in the back and limbs, and frequently nausea and retchings. The breath and respiration were uncommonly foul and offensive. The tongue in most cases was covered with a grayish white slime, through which the tongue was observed of a deep red color. The bowels more or less constipated, were generally easily moved, though sometimes they were insensible to the action of cathartics. The pulse frequent and depressed, the hands and feet cold and clammy, the skin contracted, and the general expression shrunken and haggard.

"These symptoms were ordinarily succeeded, generally in twenty-four hours, by a chill, sometimes a severe rigor, which was followed by general reaction, with frequent and bounding pulse. The chills, however, instead of subsiding, as in the accession of the

*Since the above was prepared for the press, we have received from Dr. Meeker, of LaPorte, Ia., an account of the epidemic erysipelas at that place: we will insert it in our next.—Ed.

hot stage in other fevers, were more persistent in their duration, and were frequently protracted through the continuance of the hot stage, and indeed through all the stages of the paroxysm. In some instances also, through the remissions, even embracing the whole twenty-four hours; and although the chill sometimes continued during the period mentioned, even when the body was preternaturally warm, the skin was at the same time bathed with a copious acrid perspiration.

"Another mode of attack was very different from that just described. The patient would be suddenly overtaken in apparent health and amidst his labors, with a sense of coldness, painful in the extreme, soon followed by severe chills. These symptoms were followed by pain in the head, stomach, abdomen, back and joints, or some or all these at the same time, and in the course of twenty-four or thirty-six hours, ordinarily succeeded the sore throat above mentioned. These symptoms were the principal premonitions of the subsequent efflorescence, which appeared on the skin, usually about the third or fourth day, in form of erysipelas. This efflorescence gave the qualifying characteristic of the fever, and yet in our own practice, and that of our medical associates, it did not manifest itself in more than one case in six, and when it did appear it was not confined to any particular location. Dr. Barney, of Lyman, N. H., mentions eight individuals in one family who were attacked at the same time. In each patient the disease appeared in the same locality, and travelled over the same space, involving the same surface in its progress. Usually, it was first observed on the side of the neck or face, presenting an acutely sensible and circumscribed red spot. This when first noticed might be covered with the point of the finger, but rapidly spread upwards with a defined line of demarcation on its upper margin, and in its advance embraced the whole of the face and scalp, on the side upon which it first appeared.

"Dr. Jewett to whom we have before referred, and than whom no man has had better opportunities for observing this disease, says, 'not more than one case in twenty of those having the disease as early as January or February had external erysipelas, as subsequently occurred. Occasionally, however, in the early season of the epidemic, there were severe cases of external erysipelatous inflammation affecting the head, face, body or limbs, and some cases of deep-seated cellular suppuration, pervading all parts of the system.' For the most part there was manifest swelling, tenderness and pain in the part affected, previous to the appearance of the efflorescence, and occasionally when the attack was in the face, every vestige of expression was destroyed. Frequently when the constitutional symptoms were slight, there would be extensive inflammation of the skin; and as frequently, when the external manifestation was extensive, the redness and soreness of the fauces, which preceded the attack, was uniformly modified if not entirely removed. As there was uniformly an inflamed condition of the mucous surface of the throat, preceding any constitutional disturbance of the system, and as this inflammation was usually modified or removed by the external disease of the skin; it would seem that this efflorescence upon the surface of the skin was not a symptom of the disease, but merely a translation of the inflammation from one surface to another. Often when two-thirds of one side of the body was covered with the erysipelatous inflammation, and the affection of the throat had subsided, suddenly the efflorescence would recede and the throat again become affected, and this would occur several times in one individual during the continuance of the disease."

Dr. Sutton remarks: (Am. Jrn'l Jan. '44, p. 248.)

"This disease has either assumed several characters, or we have had several epidemics traversing the county together. One was an erysipelas, connected with cynanche tonsillaris, or swelling of some of the lymphatic glands. Another was what we considered a *typhoid pneumonia*, sometimes connected with swelling of the axillary glands. These two diseases have been so intimately connected in my practice, and wherever I can hear of the epidemic prevailing, that it has been a question with me, whether the last was not a pulmonic erysipelas. The premonitory symptoms in each disease were alike; the character of the fever in each was the same; it was often the case that one form of the disease changed into that of the other; and we frequently had, in different members of the same family, the two forms of the disease at the same time. This epidemic appeared also to attack other organs, which I will notice hereafter.

"The following is a synopsis of the symptoms of this epidemic. When the throat was the part attacked, after the usual premonitory symptoms, which have been frequently mentioned, had continued for two or three days, the patient was generally seized with a chill, which lasted, in many cases, four or five hours; this was followed by a high fever, swelling of the tonsils, submaxillary, parotid, and lymphatic glands of the neck; neuralgic pains, darting over the side of the neck and head, frequently following the temporal artery; tongue, covered at first with a thick brown coat, soon became swollen and often very dark in the centre; deglutition frequently very difficult; pulse generally full, though easily compressed; skin at first hot and dry, becoming moist and continuing so after venesection. In the mild form of the disease these symptoms were frequently removed at once by an active antiphlogistic course of treatment. Sometimes the mild form had only the appearance of cynanche tonsillaris. But in the more malignant form, where the throat was affected, after the above symptoms had continued for two or three days, and sometimes from the very commencement, the pharynx became of a dark purple color; this color generally spread over the palate, tongue, and sides of the cheeks, the tongue becoming very much swollen, assuming a blackish brown color; deglutition in many cases was almost impossible. In most of these cases an erysipelas would commence at the angle of the mouth, or nose, and spread over the face and head, with all the symptoms peculiar to that disease. The inflammation of the throat was seldom stationary; sometimes passing down the trachea, with symptoms resembling laryngitis, or cynanche trachealis, and at last assuming the symptoms of pneumonia. Sometimes this inflammation passed into the nostrils, and from them into the frontal sinuses; sometimes apparently into the antrum maxillary, but in nearly every case that I saw, the *throat became well, while the erysipelas was spreading over the skin.*

"Sometimes this disease appeared to commence at the frontal sinuses and antrum; large quantities of water would be discharged from the nose, a violent pain felt over the eyebrows, or one of the malar bones, the face becoming very much swollen, the swelling closing the eyelids. These symptoms generally continued until an erysipelas made its appearance, or there was a copious discharge of bloody mucus from the nose. In the case that I met with, the neck was enormously swollen, from the left ear down to the sternum, without any redness of the skin, or but little inflammation of the pharynx; this swelling rapidly subsided, and was followed by a profound coma that terminated in death. The disease seldom presented the putrid symptoms of *cynanche maligna*, and in those cases that it did, I believe the cause might be traced to the imprudent use of mercury. In a number of cases that I met with, the inguinal glands were the seat of the disease, becoming very much inflamed, and an erysipelas first making its appearance there, and spreading over the abdomen."

Dr. Robards, of Columbia, Tenn., notices the sore throat as a symptom generally occurring, though there were cases in which the inflammation of the face, head and extremities, were unaccompanied by affection of the throat. He says, "That the disease of the throat is the same as that which attacks the skin, is evident, from the fact, that in several instances, it has extended from the former to the latter, attacking, during its progress, the submaxillary and parotid glands, and extending over the side of the face and scalp. Sometimes it descends the trachea and attacks the bronchia; at others, it attacks the pharynx and stomach. Another fact must have been observed, that when the eruption attacks the skin, the gastric and bronchial irritation at once subsides."

All the observers have noticed a tendency in the disease to attack internal organs. "In Canada, according to Dr. Colby, the disease showed itself in attacks of acute inflammation of the substance of the lungs, pleura and stomach, &c." The uterus, peritoneum mucous surface of the bladder and urethra, the external parts of generation, are all mentioned as frequent seats of the disease; extensive inflammation of the subcutaneous cellular tissue, with suppuration and disorganization to a fearful and revolting extent, is mentioned and reported by many gentlemen engaged in treating the disease.

Erratic pains of a neuralgic character, often very severe, are mentioned as usual accompanyments. Anomalous modes of attack, and various curious complications are detailed by the writers above referred to, which our limits oblige us to omit.

*(To be Continued.)*

